# Glioblastoma survival is associated with distinct proteomic alteration signatures post chemoirradiation in a large-scale proteomic panel

**DOI:** 10.3389/fonc.2023.1127645

**Published:** 2023-08-10

**Authors:** Andra Valentina Krauze, Michael Sierk, Trinh Nguyen, Qingrong Chen, Chunhua Yan, Ying Hu, William Jiang, Erdal Tasci, Theresa Cooley Zgela, Mary Sproull, Megan Mackey, Uma Shankavaram, Daoud Meerzaman, Kevin Camphausen

**Affiliations:** ^1^Radiation Oncology Branch, Center for Cancer Research, National Cancer Institute, NIH, Bethesda, MD, United States; ^2^Computational Genomics and Bioinformatics Branch, Center for Biomedical Informatics and Information Technology, National Cancer Institute, NIH, Rockville, MD, United States

**Keywords:** glioma, radiation, proteomic, genomic, classification

## Abstract

**Background:**

Glioblastomas (GBM) are rapidly progressive, nearly uniformly fatal brain tumors. Proteomic analysis represents an opportunity for noninvasive GBM classification and biological understanding of treatment response.

**Purpose:**

We analyzed differential proteomic expression pre vs. post completion of concurrent chemoirradiation (CRT) in patient serum samples to explore proteomic alterations and classify GBM by integrating clinical and proteomic parameters.

**Materials and methods:**

82 patients with GBM were clinically annotated and serum samples obtained pre- and post-CRT. Serum samples were then screened using the aptamer-based SOMAScan® proteomic assay. Significant traits from uni- and multivariate Cox models for overall survival (OS) were designated independent prognostic factors and principal component analysis (PCA) was carried out. Differential expression of protein signals was calculated using paired t-tests, with KOBAS used to identify associated KEGG pathways. GSEA pre-ranked analysis was employed on the overall list of differentially expressed proteins (DEPs) against the MSigDB Hallmark, GO Biological Process, and Reactome databases with weighted gene correlation network analysis (WGCNA) and Enrichr used to validate pathway hits internally.

**Results:**

3 clinical clusters of patients with differential survival were identified. 458 significantly DEPs pre- vs. post-treatment, 316 upregulated, 142 downregulated emerged including several pathways relevant to cancer metabolism and progression. The worst survival group (median OS 13.2 months) was associated with DEPs affiliated with proliferative pathways and distinct oppositional response (including RT) as compared to better-performing groups (intermediate, median OS 22.4 months; highest, median OS 28.7 months). Opposite signaling patterns across multiple analyses in several pathways (notably fatty acid metabolism, TNFα *via* NF-κB, Myc target V1 signaling, UV response, unfolded protein response, peroxisome, and interferon response) were distinct between clinical survival groups and supported by WGCNA. 9 proteins were statistically signficant for OS with 1 (CEACAM16) supported by KM.

**Conclusion:**

Distinct proteomic alterations with hallmarks of cancer, including progression, resistance, stemness, and invasion, were identified in serum samples obtained from GBM patients pre vs. post CRT and corresponded with clinical survival. The proteome can potentially be employed for glioma classification and biological interrogation of cancer pathways.

## Introduction

Gliomas are rapidly progressive, neurologically devastating, nearly uniformly fatal brain tumors. In glioblastoma (GBM) (WHO grade IV), the current standard of care involves maximal surgical resection followed by concurrent (CRT) radiation therapy (RT) and temozolomide (TMZ) followed by adjuvant TMZ, resulting in poor prognosis with overall survival (OS) of less than 30% at two years (1, 2). Glioma is currently classified based on morphologic appearance and molecular features while disease progression is evaluated based on clinical deterioration and imaging changes. Analysis of the proteome pre vs. post completion of standard-of-care management may allow for superior identification of outcomes and biological processes that underpin response and progression to improve personalized management and outcomes. Previous attempts at genomic and transcriptomic glioma classification have various limitations addressed by this study, including: transcriptomic analysis did not necessarily align with either proteomic analysis or clinical features (3–6), survival was not analysed in conjunction with omic data (7–9), specimen collection involved invasive approaches (e.g. tissue, CSF) and samples were not compared before and after treatment for which there is currently no data describing clinical and survival clustering with proteomic characterisation. The fact that prior proteogenomic studies have not proven effective at either prediction or prognostication and have yet to be implemented in the clinic highlights the importance of new approaches such as the one described in this study (10). Outcome based tumor stratification in glioma remains controversial as the field in general struggles to identify the optimal stratification as novel data types and findings emerge (11–13), particularly as pertaining to novel molecular markers (14) and serum proteomic approaches (15). Several studies have attempted to risk stratify GBMs based on the transcriptome (10) and/or proteome (3–7, 16); however, many studies lack sufficient clinical features to adequately stratify patient cohorts and identify confounding factors (17). A recent meta-analysis comparing GBM to normal brain (18) and a systematic review examining clinical validation results from preclinical GBM (17) identified several relevant biological pathways. Most of the molecular data is derived from the transcriptome, with a variable correlation between RNA and protein (3, 7). This is a consequence of the multifactorial sources of variability inherent in disease heterogeneity, sample acquisition and selection, approaches to data analysis, and intrinsic loose coupling between transcription, translation, and post-translational modification. The goal of this study was to generate links between proteomic alteration and clinical outcomes by uniquely analysing serum biospecimens pre vs. post therapy with the additive benefit of linkage to 10 clinical features including radiation therapy volumes with samples spanning nearly a decade 2005-2013. This study illustrates the feasibility of this approach to characterize large-scale proteomes in relationship to survival and biological signaling. We identified survival cluster specific proteomic signatures and individual protein signals that open up the possibility of treatment optimization and corresponding improvement in outcomes for GBM patients using noninvasive blood sampling.

## Materials and methods

### Patients

82 pts with pathology-proven GBM (2005-2013) enrolled on NCI NIH IRB approved protocol 04-C-0020 and treated with CRT with blood biospecimens obtained before and upon completion of CRT were included in the study. 29 patients in this cohort also received concurrent valproic acid (VPA) on NCI NIH IRB approved protocol subject to separate analysis. Serum samples were screened using the multiplexed, aptamer-based approach (SomaScan^®^ assay) in the SomaLogic^®^ research facility with the process carried out by the Somalogic^®^ group using samples provided by the authors to measure the relative concentrations of 7596 protein targets (7289 human) for changes in expression using approximately 150 ul of serum (19, 20). Clinical data (age, gender), tumor characteristics (location, MGMT methylation status), management-related factors (extent of resection), RT volumes (GTV T1, GTV T2), recursive partitioning analysis score (RPA) (21), and outcomes (PFS, OS) were obtained or derived (RPA) from the electronic health record with GTV T1, GTV T2, generated per ICRU report 83 (22) obtained from the RT treatment planning (contoured on the T1 gadolinium and T2 FLAIR (Fluid attenuated inversion recovery) sequences respectively of the MRI scan employed for RT planning per standard guidelines). The clinical and proteomic dataset query and storing operations were provided by NIDAP (23).

### SomaLogic^®^ SomaScan assays

Serum samples were obtained before initiation (average 7 days, range (0 to 23)) and following completion of CRT (average 7 days, range (-1 to 30)), with the time between pre and post sample acquisition averaging 49 days (range 27- 83 days). Following acquisition, samples were frozen at -80° for an average of 3442 days (range 800 -5788 days or 2.2 – 15.9 years) and then defrosted and screened using the aptamer-based SomaScan^®^ proteomic assay technology from SomaLogic^®^ for changes in expression of 7596 protein analytes (19, 20). SomaScan^®^ data was filtered to any aptamer-based SomaScan^®^ proteomic assay targets representing non-human organisms and non-human proteins or controls (i.e., Non-Human, Spuriomer, Hybridization Control Elution, Non-Biotin, Non-Cleavable, Spurimer) from 7596 protein analytes. Total 7289 unique Somalogic^®^ proteomic assay aptamers targeting 6,386 unique gene symbols were retained for analysis. Normalized RFU values reported by SomaScan^®^ were log2 transformed.

*Statistical Analysis*

### Cox proportional hazards regression models for survival

Univariate Cox overall survival (OS) models were generated using the coxph function from the R survival package (24) for each available clinical trait. Traits with p-value < 0.05 (age, MGMT methylation status, GTV T1, RPA score, and VPA administration status) were selected to be used in a multivariate Cox regression analysis. The variables with a final p-value < 0.05 (age, given a binary classification into < or >= 50 years old, MGMT status, and GTV T1) were designated as independent prognostic factors.

To identify subgroups among the cases, we used following three steps of analysis. First, we performed principal component analysis using the significant independent prognostic factors. Second, we selected the principal components that are associated the most with overall survival time. Finally, we used the selected principal components to cluster the patients using the partitioning around medoids (pam) function (25) in the R cluster package with parameters metric = “euclidean” and k=3”.

We used the R modules Survfit and coxph to perform OS analysis based on the three resulting subgroups from the clustering analysis and MGMT status (whole cohort and within the resulting subgroups) (24, 26).

### Proteomic pathway signatures

We performed a paired t-test using post- vs. pre-CRT SomaScan^®^ RFU (Relative Fluorescent Units) values and calculated false discovery rate (FDR) values using the Benjamini-Hochberg method. Significantly upregulated (Log2FC >= 0.2, FDR < 0.05) and down-regulated (Log2FC <= -0.2, FDR < 0.05) proteins were entered into the KOBAS server 2.0 (27) to identify associated KEGG pathways and visualize them *via* bubble plot. GSEA version 4.2.3 from the Broad Institute (28, 29) was used to carry out pre-ranked analysis on the overall list of differentially expressed proteins, with the ranking based on the t statistic from the paired t-test, against the MSigDB Hallmark (h.all.v2022.1.Hs.symbols.gmt), GO Biological Process (c5.go.bp.v2022.1.Hs.symbols.gmt), and Canonical Pathways (c2.cp.v2022.1.Hs.symbols.gmt) gene sets. (**Supplementary File**). Pathways were considered significant if the FDR for the Normalized Enrichment Score was < 0.25.

### Association with clinical traits *via* survival subgroups

To combine the protein measurements post- vs. pre-CRT within the subgroup into a single readout as input for ssGSEA2.0 (30), a paired t-test using t.test function in R with parameter paired=TRUE was employed, and the resulting vector of t values was used as input for ssGSEA2.0. The t values were first rank-normalized (sample.norm.type = “rank”). The rank-normalized profiles were further Z-scored (correl.type = “z.score”). The weight parameter was set to 1 (weight =1) to incorporate the t values into the calculation of enrichment scores and p values for each signature across the three subgroups individually against the MSigDB Hallmark gene sets by downloading the gmt files of MsSigDB collections hallmark (28) and KEGG signatures version V2022.1, from http://www.gsea-msigdb.org/gsea/msigdb/collections.jsp. Lastly, gene sets with Benjamini-Hochberg FDR < 0.05 in at least one patient subgroup were selected to visualize normalized enrichment scores (NES) in a heatmap to show the overall pathway enrichment.

### Survival associated protein signal analysis

Univariate Cox modeling using the post-pre log2-transformed RFU values was performed to identify proteins associated with OS. ANOVA was performed to identify differentially expressed proteins (DEPs) between the three clinical subgroups with 9 significant proteins (*p* < 0.05) in both tests. KM analysis on these 9 proteins was performed using the survminer package by separating patients into two groups with log2FC greater than or less than zero.

### Functionally related proteins and clinical associations

Using the matrix of difference between post-pre CRT log2 transformed RFU values as an input, we applied WGCNA (31), an R package for weighted gene correlation network analysis, to detect the modules associated with the three resulting subgroups, the overall clinical variables, and survival data. We generated a signed network with corType = ‘bicor’ and softthreshold=16. Genes associated with modules significantly associated with clinical traits (*p* ≤<= 0.05) were used to do pathway enrichment analysis using Enrichr (32).

## Results

### Patient cohort and captured clinical features

Eighty-two patients, with a mean age of 56 (range 29-79), 73% male, 66% with cortical disease, a reported resection, and tumor methylation status, with pre and post CRT serum samples, were included in the analysis (**Table 1**). Twenty-nine of the patients also received concurrent valproic acid (VPA) in addition to concurrent temozolomide and radiotherapy (RT) on protocol (the effect of VPA is analyzed and reported on separately). RT volumes in the form of gross tumor volume (GTV T1) and GTV T2 were captured from the RT treatment planning system (**Table 1**), with the majority of patients treated with an intensity modulated technique (71%).

**Table 1:** Demographic and clinical variables for the patient cohort (n = 82).

Clinical Variables				
Age (years)				
	mean	median	range	
	56.04	56.50	29-79	
Gender, (%)				
	Male	Female		
	60 (73.2%)	22 (26.8%)		
Location, (%)				
	cortical	periventricular		
	54 (65.9%)	28 (34.1%)		
Extent of resection, n (%)				
	GTR	STR	Biopsy	
	30 (36.6%)	45 (54.9%)	7 (8.5%)	
MGMT, n (%)				
	methylated	unmethylated	unknown	
	21 (25.6%)	31 (37.8%)	30 (36.6%)	
RPA, n (%)	3	4	5	Unknown
	14 (17.0%)	46 (56.0%)	19 (23.0%)	3 (4.0%)
Radiation therapy volumes				
GTV T2 (cm3) (%)				
	<10	10-50	50-100	>100
	8 (9.8%)	21 (25.6%)	23 (28.0%)	30 (36.6%)
GTV T1 (cm3) (%)				
	<20	20-40	>40	
	24 (29.0%)	28 (34.0%)	30 (37.0%)	
Radiation technique, n (%)				
	Arc	IMRT	30	
	20 (24.4%)	38 (46.3%)	24 (29.3%)	
VPA (Valproic acid) n(%)	Male	Female		
No	39 (48%)	14 (17%)		
Yes	21 (26.0%)	8 (10.0%)		

### Clustering of patients using clinical factors results in survival groups based on age, MGMT methylation status and GTV T1

**Figure 1:**
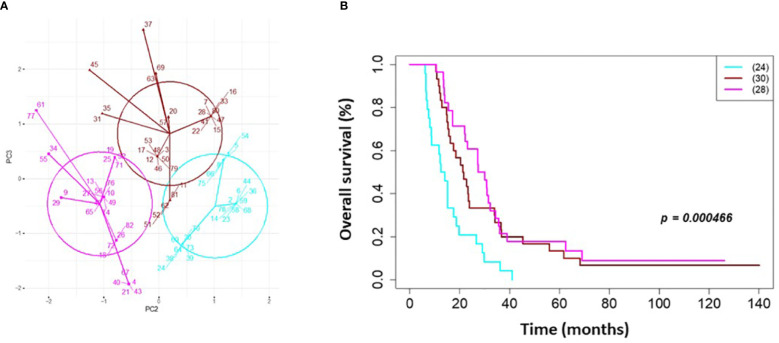
Clinical clustering. Patient clustering based on clinical features associated with overall survival. **(A)** Principal component analysis cluster plot based on PC3 and PC2 correlated with overall survival time based on age, MGMT methylation status, and GTV T1 with Cox proportional hazard p-values < 0.05. Circles represent clusters of patients (n=82). A point with a number indicates the patient study ID. **(B)** Overall survival by clinical cluster. Cyan = lowest survival. Purple =intermediate survival. Pink = highest survival.

**Figure 2:**
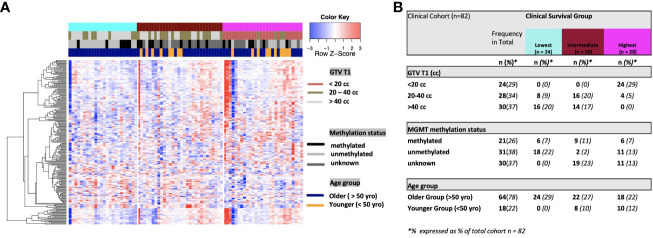
**(A)** Heat map of clinical clustering and proteomic signal following overall survival analysis based on GTV T1, MGMT methylation status, and age group with Cox proportional hazard p-values < 0.05. The dendrogram represents 221 significantly expressed proteins pre- vs. post-treatment. Unknown methylation status is represented by the color light grey. **(B)** Frequencies of clinical factors in whole cohort and clinical survival group (GTV T1, MGMT methylation status and age group) broken down by survival group n (expressed as % of total cohort n =82).

**Figure 3:**
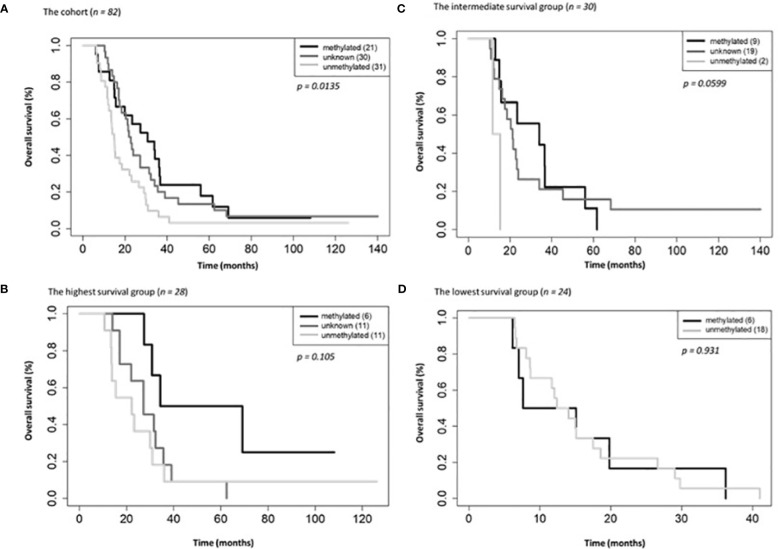
Overall survival by MGMT methylations status for the entire cohort **(A)**, the highest **(B)**, intermediate **(C)** and lowest **(D)** survival groups.

On univariate and multivariate Cox analysis for clinical independent prognostic factors (**Table 2**), age group (> vs. <= 50 years old) (*p* < 0.006), MGMT methylation status (*p* = 0.009), and GTV T1 (*p* = 0.007) were associated with OS (**Table 2**). These clinical features were included in the clinical clustering of patients (**Figure 1**) with three clusters of patients with differential survival (Kaplan-Meier log-ranked p-value = 4.66x10^-6^) identified with the lowest (subgroup 1 (cyan), n=24), intermediate (subgroup 2 (maroon), n=30) and highest survival (subgroup 3 (magenta), n=28) exhibiting median survival of 13.2, 22.4, and 28.7m, respectively (**Figure 1**). The subgroups were clinically distinct. When comparing the GTV T1 variable the highest survival group was made up exclusively of patients with smaller GTV T1 volumes (< 40 cc, with all the patients with volumes of <20 cc present in this group). Age also proved a factor for survival with the poorest survival group solely comprised of patients over the age of 50. MGMT methylation status was mixed across the groups, with the lowest survival group having a more significant proportion of MGMT unmethylated patients (75% unmethylated), the intermediate group having the most significant proportion of unknown MGMT status (77%), and the highest surviving group having equal numbers of unmethylated and unknown status patients. (**Figure 2**). Consistent with the Cox model, MGMT status was statistically significant in the KM analysis (p=0.0135) with unmethylated, unknown, and methylated patients having median OS of 15, 22.4, and 30.8 months (**Figure 3A**). However, within the survival groups, MGMT status was not statistically significant (**Figures 3B–D**).

**Table 2:** Univariate and multivariate Cox analyses for overall survival for clinical independent prognostic factors.

Univariate and multivariate Cox proportional hazards regression analysis								
Characteristics	Univariate				Multivariate			
	HR	lower 95	upper 95	p value	HR	lower 95	upper 95	p value
Age group								
Older (>50 yro)	Reference							
Younger Group	0.446	0.25	0.794	0.006	0.236	0.068	0.814	0.022
Gender								
Female	Reference							
male	1.539	0.911	2,601	0.107				
Location								
Cortical	Reference							
Periventricular	1.53	0.951	2,463	0.08				
Extent of Resection								
Biopsy	Reference							
GTR	1.391	0.63	3,074	0.414				
STR	1,647	0.768	3.531	0.2				
MGMT methylation status								
methylated	Reference					Reference		
unmethylated	2.184	1.215	3,925	0.009	2.375	1.264	4,465	0.007
unknown	1.198	0.667	2.151	0.546	0.907	0.482	1.707	0.762
RPA								
3	Reference				Reference			
4	2.166	1.11	4.227	0.023	0.521	0.133	2,033	0.348
5	1.745	0.832	3.66	0.141	0.25	0.054	1.146	0.074
Unknown	9.791	2.553	37.546	0.001	1.742	0.259	11.734	0.569
Radiation therapy volumes								
GTV T2 (cc)								
Coded <10cc	Reference							
Coded 10-50cc	0.523	0.219	1.251	0.145				
Coded 50-100	0.528	0.221	1.263	0.151				
Coded >100 cc	0.814	0.355	1.864	0.626				
GTV T1 (cc)								
Coded <20 cc	Reference				Reference			
Coded 20-40cc	1.47	0.828	2.61	0.188	1.393	0.772	2.513	0.271
Coded >40	2.183	1.241	3,841	0.007	2.904	1.572	5.366	0.001
Radiation technique								
3D	Reference							
Arc	1.079	0.579	2.01	0.811				
IMRT	0.951	0.562	1.61	0.852				
VPA								
No	Reference				Reference			
Yes	0.584	0.362	0.941	0.027	0.664	0.386	1.143	0.139

IMRT (Intensity Modulated Radiation Therapy), MGMT (O6-methylguanine-DNA methyltransferase), GTR (Gross Total Resection), STR (Subtotal Resection), GTV (Gross Tumor Volume), RPA (Recursive Partitioning Analysis), VPA (Valproic Acid).

### Differential protein expression in serum following CRT reveals pathways relevant to cancer both up and downregulated

**Figure 4:**
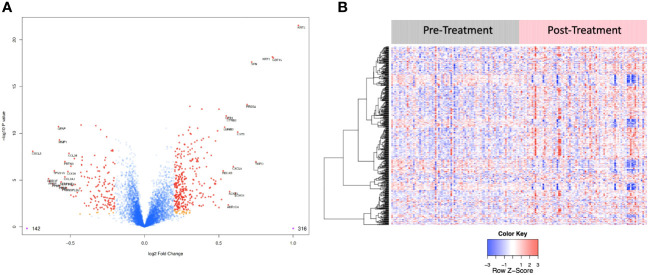
**(A)** Volcano plot of differentially expressed proteins pre vs. post treatment. Red dots represent the significant proteins that passed cut-off (|Log2FC| > 0.2 & FDR < 0.05) (458 proteins). Orange dots represent the poteins with |Log2FC| > 0.2 but not FDR < 0.05. Blue dots represent proteins that are not significant (i.e., did not pass either threshold). 316 proteins were up regulated, and 142 proteins were downregulated. The top 15 proteins that decreased in value (left aspect of plot) and those that increased in value (right aspect of plot) based on |FC| were labeled with the identified proteins’ names. **(B)** Heat map representation of the expression levels of selected, differential expressed proteins (N=458) pre vs. post treatment.

**Figure 5:**
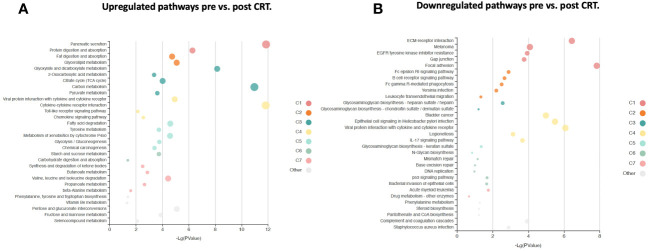
KOBAS bubble plot of KEGG pathways enriched in the set of significantly differentially expressed proteins between pre- and post-treatment based on a paired t-test. **(A)** Upregulated genes (FDR < 0.05).. **(B)** Downregulated genes (FDR < 0.05).. The bubble size reflects the KOBAS hypergeometric test p-value broken into the following ranges (smallest to largest): [0.05,1], [0.01,0.05), [0.001,0.01), [0.0001,0.001), [1e-10,0.0001), [0,1e-10). Colors reflect clusters of related pathways. Clusters of pathways are determined by creating edges if the Jaccard Index between pathways is larger than 0.35 and then clustering using the Infomap algorithm.

6,386 human proteins were analyzed pre- vs. post-CRT using the 7k SomaScan® proteomic panel. The time the samples were stored in the freezer from collection to analysis did not reveal any systematic impact on protein measurements, as shown in (**Supplemental Figure 1**). We performed a paired t-test on the post- vs. pre-CRT log2-transformed SomaScan^®^ RFU values to identify significantly differentially expressed proteins between pre-and post-treatment. The log2-fold change values ranged from 1.04 to -0.75, with 2298 proteins having an FDR less than 0.05 with 458 proteins having an |Log2FC| >= 0.2 (316 upregulated, 142 downregulated, **Figure 4**). The significantly up- and downregulated genes were entered into the KOBAS server, which performs gene set enrichment analysis using the hypergeometric test and generates plots (27). **Figure 5** shows that pathways relevant to cancer, such as the Ras, MAP kinase, and NOTCH signaling pathways and various metabolic pathways, are upregulated post CRT according to KOBAS. Overall various metabolic pathways are upregulated, and immune-related pathways are downregulated. The colors in **Figure 5** are clusters of pathways with overlapping genes. The fact that multiple related pathways are up or downregulated together suggests that the differential expression measures a meaningful biological signal and is not artifactual. Some pathways such as PI3K-Akt and Toll-like receptor signaling pathways are both up and downregulated reflecting heterogeneous cell composition and differential response to CRT. These results were confirmed using the pre-ranked GSEA method with the t-statistic for all proteins from the paired t-test against the MSigDB Hallmark, Canonical Pathways, GO Biological Process gene sets, and many of the same pathways were determined to be significant (**Supplemental File**). The pathways upregulated, such as Ras/MAPK/PI3K/AKT, metabolism, Notch signaling, and axon guidance, are consistent with previous proteomic markers of GBM, ie. active tumor including the presence of stem cells. The pathways that were downregulated after therapy, including TNFα or TGF-beta, are consistent with response to radiation and chemotherapy representing protein release from dying cells and residual/proliferating tumor cells. This demonstrates that the serum proteome is altered after CRT in GBM patients.

### GBM survival groups are associated with differential signaling pathways and serum protein expression

**Figure 6:**
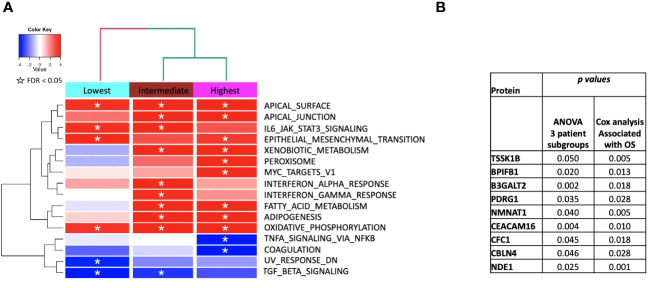
**(A)** ssGSEA2.0 associations with Hallmark pathways for proteins differentially expressed within patient subgroups. Paired t-tests were run within each of the patient subgroups from **Figure 1**. These results were fed into ssGSEA2.0 and significant pathways (FDR < 0.05) in at least one of the three subgroups were selected. The heatmap shows the GSEA normalized enrichment score. **(B)** The 9 proteins statistically significant across the three subgroups (p value (ANOVA) < 0.05) and associated with OS in Cox analysis (p < 0.05). Kaplan Meier analysis for statistically significant proteins is shown in **Supplemental Figure 1**.

We then performed gene set enrichment analysis on the patient subgroups using ssGSEA2.0 with rankings based on the t-statistic from paired t-tests carried out within each subgroup. **Figure 6** shows the identified MSigDB Hallmark pathways with an FDR < 0.05 (**Figure 6A**), including Apical Junction, IL-6/JAK/STAT3 Signaling, Epithelial Mesenchymal Transition, Xenobiotic Metabolism, Fatty acid metabolism, TNFα signaling *via* NF-κB, Interferon alpha and gamma response, UV response down. Some signal alterations are shared amongst the groups, specifically epithelial-mesenchymal transition (EMT)(elevated), oxidative phosphorylation (elevated), and TGFbeta *via* NF-κB (decreased). The patient subgroups have distinct pathways that are up or downregulated, indicating the potential to characterize patient subgroups based on their serum protein expression. The lowest survival group lacks associations with metabolic pathways that distinguish intermediate and higher survival groups (**Figure 6A**). By contrast, the highest survival group exhibits a reduction in TNFα signaling and an increase in Peroxisome and Myc targets V1. The intermediate survival group shares similarities in the direction of expression, with the highest survival subgroup significant for xenobiotic metabolism, apical surface and apical junction, adipogenesis, and fatty acid metabolism. The intermediate subgroup is distinctly significantly associated with interferon-alpha and gamma response, glycolysis, and UV response UP. Similar associations with pathways in cancer were noted when examining enriched KEGG pathways (**Supplemental Table 1**). Statistically significant oppositional signaling was identified in fatty acid metabolism, glycolysis gluconeogenesis, and propanoate/butanoate/xenobiotic metabolism (elevated in the best survival group and decreased in the lowest survival group). Focal adhesion, PPAR signaling, and lysine degradation were notably reduced in the lowest survival group. Interestingly some pathway directionality was distinct in KEGG vs. Hallmark (e.g. e.g., ECM for the intermediate group was up in Hallmark and down in KEGG), indicating that pathway directionality needs to be interpreted with caution (**Supplemental Table 1**). Overall superior-performing groups shared considerable similarities in both ssGSEA and KEGG following the administration of CRT, particularly concerning metabolic pathways (elevated) and DNA repair (decreased).

**Figure 7:**
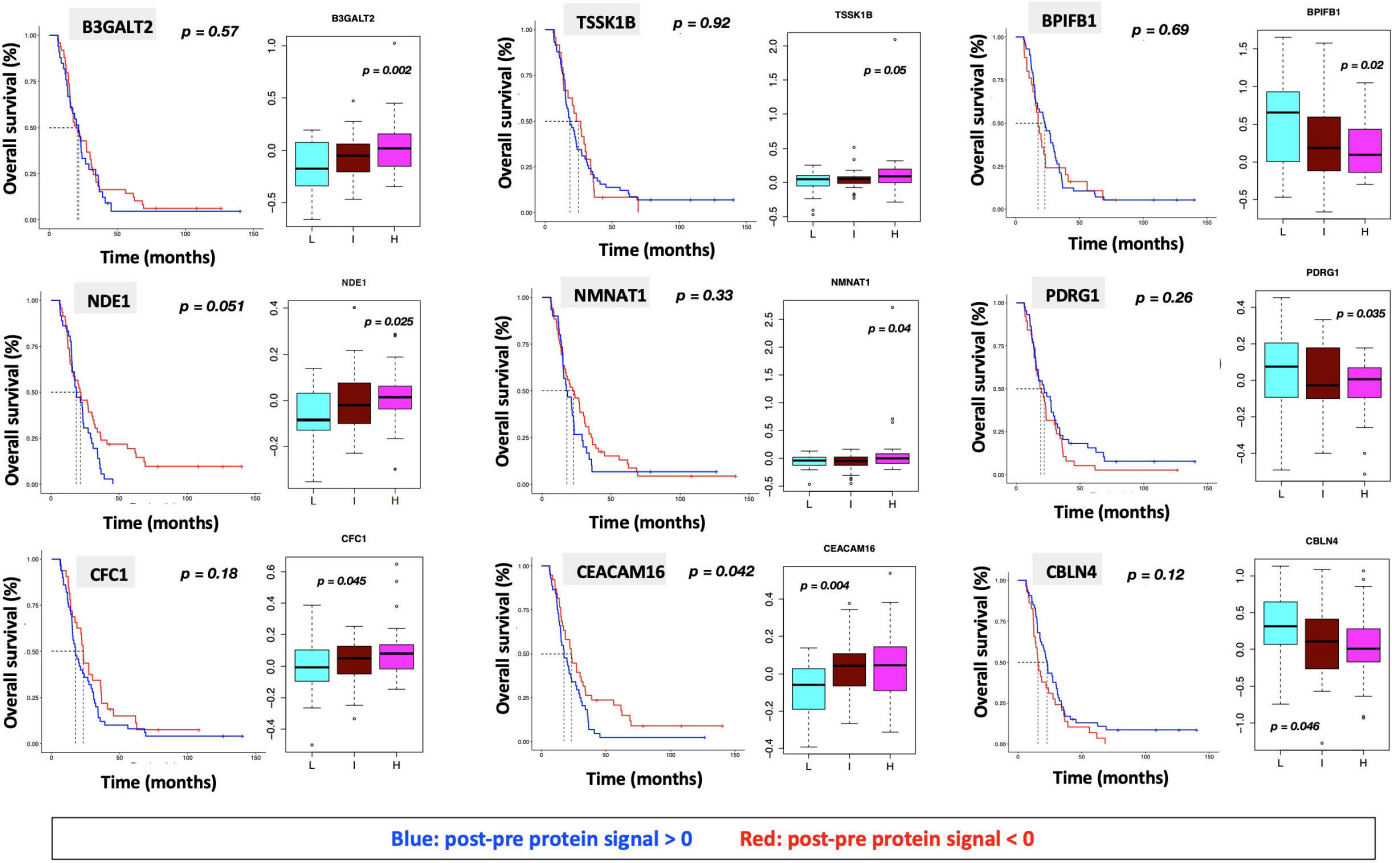
Overall survival analysis for 9 proteins statistically significant across the three subgroups (p value (ANOVA) < 0.05) and associated with OS in Cox analysis (p < 0.05) with associated protein signal box plots.

We identified 9 proteins that were statistically significant across the three subgroups *via* ANOVA (p-value < 0.05) and also associated with OS in a univariate Cox model (p < 0.05) (**Figure 6B**). Five of these were also statistically significant upon Kaplan-Meier analysis (NPS, NETO2, SEMA6D, CBLN4, CST7) (**Figure 6B**). One was also statistically significant upon Kaplan-Meier analysis (CEACAM16). These nine proteins all have plausible connections to relevant pathways; however, box plots of their expression demonstrate the significant overlap between patient subgroups, indicating they are unlikely to be effective as prognostic indicators. (**Figure 7**). Paired t-tests within the clinical subgroups (top 10 proteins emerging show in **Table 3**) reveal distinct proteins between the survival groups with respect to fold change and p values with proteins present in all groups (KRT5, KRT1, SFN, GDF15) and others (MGMT in lowest survival group). Overall most of the proteins are elevated post treatment as compared to prior to treatment and while p values are statistically significant, FDRs are only significant for the top 2 proteins in the lowest survival subgroup while highly significant in the intermediate and high survival subgroups (**Table 3**).

**Table 3:** The top ten proteins based on p-value identified in a paired t-test comparing pre- and post-CRT expression for each patient subgroup.

Lowest Survival Subgroup (n=24)			Intermediate Survival Subgroup (n=30)			Highest Survival Subgroup (n=28)		
Symbol	p-value	Log2FC	Symbol	p-value	Log2FC	Symbol	p-value	Log2FC
MGMT	7.29E-06	-0.627	*SFN*	2.74E-11	0.808	**KRT5**	9.83E-10	1.128
**KRT5**	1.04E-05	0.986	**KRT5**	8.01E-10	0.993	**KRT1**	4.70E-08	0.996
GDF15	3.03E-05	0.638	**KRT1**	1.80E-09	0.827	*SFN*	2.40E-07	0.775
EDA2R	3.34E-05	0.176	**GDF15**	4.59E-09	0.980	**GDF15**	3.26E-07	0.944
GFAP	3.56E-05	-0.681	FAS	7.64E-09	0.339	**SERPIND1**	2.73E-06	-0.264
**KRT1**	1.14E-04	0.749	CPB1	3.14E-08	0.781	**ACP7**	4.27E-06	-0.213
ILR1	1.21E-04	0.375	*PRSS1*	3.53E-08	0.516	**HMX2**	4.94E-06	-0.085
TAGLN	1.60E-04	0.318	CTRB2	8.42E-08	0.689	**GUCA1B**	5.45E-06	-0.171
*PRSS1*	1.93E-04	0.302	CBR3	9.27E-08	0.313	**PRSS27**	6.00E-06	-0.264
PRSS2	3.02E-04	0.518	BOC	1.23E-07	0.587	**RAB26**	6.19E-06	-0.171

Gene symbols appearing in all three subgroups are shown in bold, those appearing in two subgroups are shown in italics. Genes symbols whose expression dropped post treatment are underlined. FC, fold change.

### Association of clinical characteristics and survival groups with differential protein expression in serum reveals module clinical trait relationships correlating with progression free and overall survival

**Figure 8:**
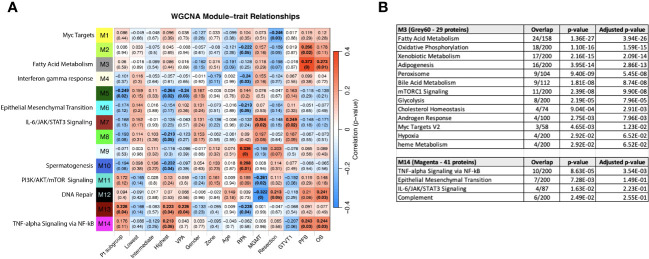
**(A)** WGCNA Protein module-clinical trait relationships heatmap for post-pre data with clinical features. The WGCNA protein modules are labeled with the MSigDB Hallmark pathways with the lowest adjusted p-value according to Enrichr. The M3 (Grey60) and M14 (Magenta) modules significantly correlate to overall survival and progression free survival. M7 (Brown) significantly correlated with MGMT methylation status and GTVT1. The top numbers indicate correlation coefficients, numbers in brackets indicate p-values. Bold indicate cells with p ≤ 0.05. **(B)** GO Biological Processes that are associated with the M3 and M14 modules according to Enrichr. MGMT methylation status (1= methylated, 2= unmethylated).

WGCNA was performed to identify groups of similarly-expressed genes (referred to as modules) significantly correlated with clinical traits (31). The most significant associations with OS and PFS were with modules M3 (Grey60) and M14 (Magenta) (**Figure 8A**). The GO biological processes associated with the M3 and M14 modules, are shown in **Figure 8B**. The grey60 (M3) and magenta (M14) modules contain genes associated with angiogenesis and cell proliferation via KRAS signaling down. The dark turquoise (M6) module contains genes associated with angiogenesis and cell proliferation *via* KRAS signaling down. Several of the same biological pathways (e.g., IL6/JAK/STAT signaling, KRAS signaling, EMT) were identified using both the differential expression between subgroups and WGCNA approaches, suggesting that we are observing genuine signals and not an artifact of any particular method. However, it is important to note that the correlation coefficients between the modules and clinical features are relatively low, with the absolute value ranging from 0.20 to 0.33. This is to be expected looking for signals from brain tumors in the blood, but it does caution against overinterpretation of the results and may reflect proteome functionality in a separate dimension with a loose connection to clinical factors.

Given that the survival groups represent aggregates of clinical factors, we wanted to determine if their interaction with the WGCNA modules is more robust compared to individual clinical characteristics and compare this to pathways identified in the previous analyses. To determine if the subgroup and WGCNA methods were detecting the same signals, we created dummy variables for the subgroups, collectively as group “Pt subgroup” in **Figure 8A**, and individually with patients in the “Lowest”, “Intermediate”, and “Highest” in **Figure 8A** being associated with the dummy variables 1, 2 and 3, respectively. Consistent with **Figure 6A**, both “Pt subgroup” and “Highest” are significantly correlated with modules M5 (Dark green) and M13 (Dark Red) while the “Highest” subgroup is associated with module M14 (Magenta) which is enriched for TNF-alpha Signaling via NFkB, Epithelial Mesenchymal Transition, IL-6/JAK/STAT3 Signaling, Complement, Apical Junction, Xenobiotic Metabolism, Apoptosis, Unfolded Protein Response, KRAS Signaling Up and UV Response Up. The opposite sign of the correlation is due to the lowest survival group having the smallest number in “Pt subgroup”, but the higher number (1 vs 0) in the “Lowest” variable. The highest survival subgroup has an inverse relationship with module M8 (tan), which is associated with TNFα signaling *via* NF-κB (again similar to **Figure 6A**), and a similar relationship is seen with VPA administration. The highest survival subgroup is enriched in the proportion of VPA treated patients (14/28) (50%) vs. subgroup 1 (5/24) (21%) and subgroup 2 (10/30) (33%). Modules 11 (PI3K/AKT/mTOR signaling) and 12 (DNA repair) are negatively associated with MGMT status (with a low correlation coefficient of -0.26 and -0.32 respectively) and positively associated with M7 (Brown - which is associated with MGMT status and GTVT1), with a correlation coefficient of 0.25. The Grey60, Magenta and Brown modules and their Enrichr results are available as Supplemental files. As mentioned above MGMT status only imperfectly predicts survival, and thus identifying proteins weakly associated with MGMT status is unlikely to generate a prognostic signal. By including the patient subgroups in the WGCNA analysis, both collectively and individually, each subgroup was significantly associated with a different module, again indicating differential protein expression between the subgroups and lending further support that a genuine signal results from the alteration in the proteome pre vs. post CRT. The findings point to further investigation that can be done with significant module-trait relationships to uncover potentially meaningful biological correlates.

## Discussion

This study is unique in examining a population of patients with GBM that received CRT and underwent large-scale 7k serum proteomic analysis prior to and after treatment. The novelty of this study involves several critical aspects that have not been described elsewhere to date: 1) the provision of non-invasive analysis using serum at two time points in GBM; 2) the aggregation with 10 clinical features including radiation therapy volumes; 3) The collection and effective analysis of serum samples collected pre and post treatment spanning nearly a decade 2005-2013 illustrating the feasibility of this approach; 4) the characterization of the 7k proteome on an aptamer platform linked to survival and biological signaling. This setup uniquely allows for analysis of clinical features and proteomic alterations. Based on existing evidence we have no clear resolution on which clinical or outcome variables constitute “ground truth” as pertaining to the proteome. Given that the largest databases of outcomes originate in the Radiation Therapy Oncology Group (RTOG), we elected to employ clinical features that the RTOG uses acknowledging also that the RTOG does not currently have outcomes with IDH status nor microarray grouping. The goal was to frame the alteration in the proteome in the most robust manner possible and since all clinical features suffer from limitations in both acquisition and capture we determined that overall survival was the most robust parameter that would in its current state stand the test of time. The interpretation of what and when constitutes progression in glioma remains highly controversial and is actively evolving as imaging progression in glioma is subject to Response Assessment in Neuro-Oncology (RANO) criteria themselves adapting to both novel imaging analysis and the addition of novel agents (33).

The analysis of clinical variables in relationship to survival revealed results similar to published literature, with the best-performing patient subgroup exhibiting a median OS of 28.7 months and the worst-performing group 13.2 months (34–38). Patients were stratified using three factors previously identified as prognostic for GBM: age (21, 39), MGMT (40), and GTV T1 volume (41). These factors represent different facets of the patient-disease-treatment matrix: age to patient physiology, morbidities, and tumor biology; MGMT to chemotherapy response (42); and GTV T1 to radiation dosage (41). GTV T2 was not significant despite it (more specifically T2 FLAIR signal) having been associated with OS in glioma and representing a possible surrogate for tumor load (43); in this cohort, GTV T2 may represent an insufficiently robust parameter when captured by clinical contouring for RT planning as compared with its capture and analysis *via* MRI imaging sequences.

### Pathway analysis

**Figure 9:**
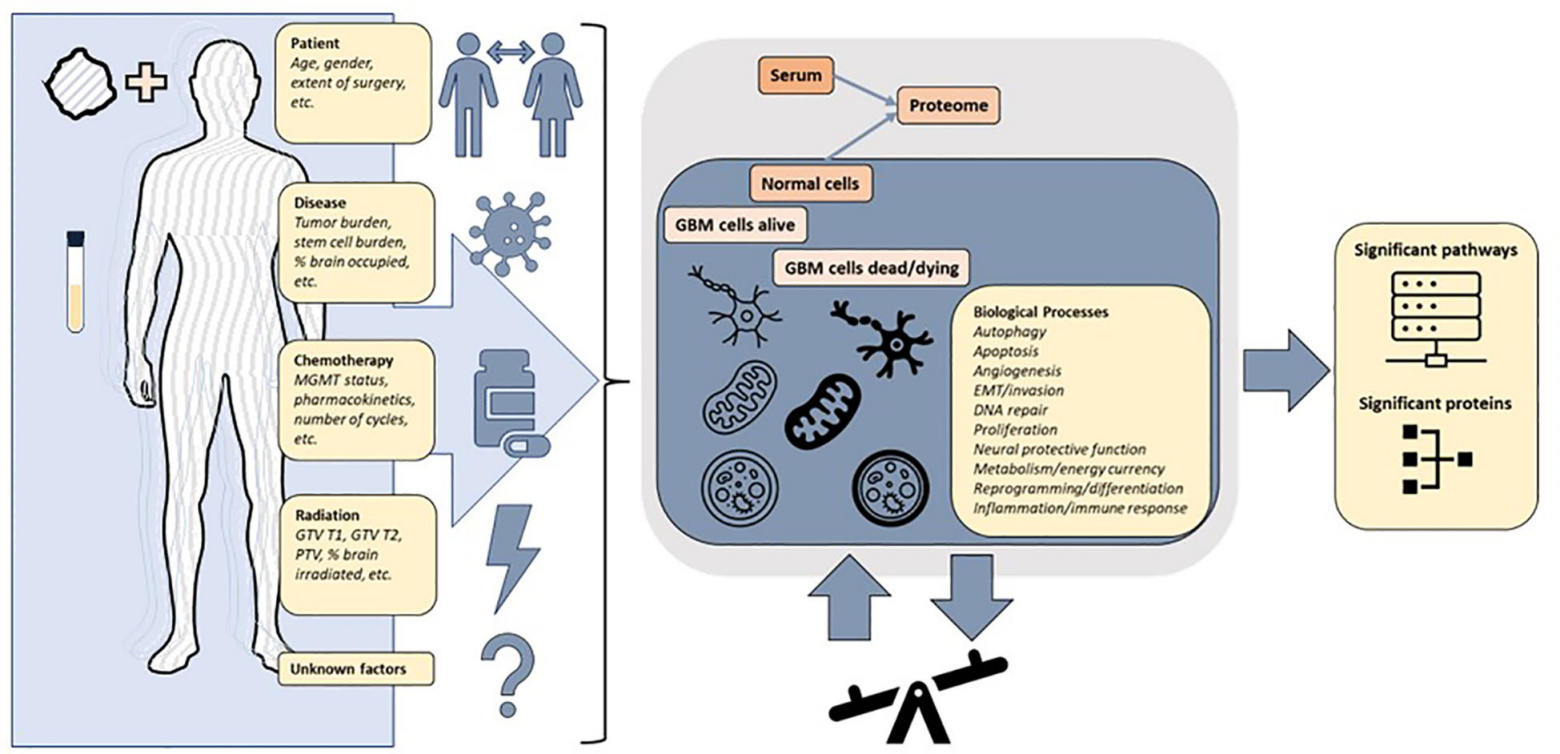
The protein signals in a patient with glioblastoma are reflective of a combination of factors including patient and tumor heterogeneity pre and post treatment. The changes in expression of proteins and pathways measured in the blood reflect a superposition of multiple overlapping signaling pathways. The underlying biological processes can be inferred from differentially altered pathways and proteins, but further precise multimodal measurements are required to achieve clinically actionable understanding of patient and tumor response to treatment.

Given redundant signaling, variation over time, variation between individuals and most importantly the measurement of protein signals reflecting a composite of the whole patient, the tumor and all interventions, it is not self-evident that a serum signal from a brain tumor should be detectable. It is important to emphasize that known and unknown factors result in signals measured in serum (**Figure 9**). In this study, we demonstrated that the serum proteome in a 7K panel was significantly altered pre vs. post completion of CRT in GBM, a finding that has not previously been described. The RT response itself involves direct and indirect effects with different types of cell death and immune responses persisting days to weeks following RT (44). Given that the post protein signal is based on protein signals captured anywhere from -1 to 30 days after RT, in conjunction with patient and tumor heterogeneity, significant variability is further expected. The evolution of individual protein signals over time is still being determined, especially on this scale and in acquisition *via* serum (45). Additionally we were able to show that signal alteration exists in serum with similar findings generated *via* different analyses, hence it is realistic to pursue proteomic analysis of serum sample alteration to examine GBM tumor response, progression, transformation, and outcome. The alterations identified are consistent with biological pathways previously described in cancer and glioma, including angiogenesis, proliferation, stemness, metabolism and immune response (3, 15, 18, 46) (**Supplemental Figure 2**). The clinical subgroups exhibit distinct proteomic signatures consistent with known risk factors for GBM progression. The group with the lowest survival, distinguished by UV_response_DN and a lack of association with metabolic pathways observed in the intermediate and higher survival groupsin the Hallmark genesets, all similarly noted in KEGG and theWGCNA analysis, reveals a pathway signature consistent with poor prognosis in glioma *via* increased proliferation, hypoxia, enrichment in glioma stem cells, and radiation resistance *via* Notch signaling (47), IL-6_JAK_STAT3 (48, 49). Pathways that are less intuitive such as Heme_metabolism include BMP-2-inducible protein kinase (50) described as promoting adaptation of GBM cells to glucose starvation, MAP2K3 linked to poor clinicopathologic features and negatively correlated with prognosis in glioma (51) and HDGF previously implicated as a serum marker of cancer progression (52). Thus the lowest survival group with median survival of just over a year is distinguished by the pathways that are consistent with rapid recurrence and treatment resistance that is clinically observable in some patients and now revealed in the serum proteome.The highest survival group by contrast has a proteomic pathway signature with unique associations involving increased peroxisome and Myc targets v1 signaling and a decrease in TNFα signaling *via* NF-κB. These pathways suggest a differential response to therapeutic intervention involving both RT and chemotherapy and an interplay with immunometabolism. Peroxisomes are involved in lipid metabolism and cellular redox balance and are central to fatty acid oxidation as well as reactive oxygen species homeostasis and cancer immunometabolism (53), all of which bear a connection to pathways in this analysis given that fatty acid metabolism was elevated in both intermediate and high survival groups but noticeably absent in the lowest survival group. The peroxisome hallmark geneset notably includes IDH1 and 2, SMARCC1 (also part of Myc targets v1), transcription factors part of PD1 target associated with survival and immune signaling in glioma (54). The decrease in TNFα signaling *via* NF-κB and the increase in peroxisome in the best-performing patients could indicate a possible relationship to glioma stem cell burden, tumor invasion, and RT response (55, 56) resulting in a more favorable outcome (**Supplemental Figure 2**). Interestingly, the superior and intermediate performing groups share several similarities in metabolic pathways (xenobiotic metabolism, fatty acid metabolism, adipogenesis) as well as apical surface and apical junction, all of which were not identified as altered in the poorer performing patients enforcing the hypothesis of a fluctuating balance of powers in the interplay of pro and anti stem cell and resistance signaling between these two extremes of survival, equally mirrored by pathways that are elevated (EMT, oxidative phosphorylation) or decreased (TGF beta), with differential behavior depending on the tumor context (57, 58)).

With respect to the impact of therepeutic interventions that may be exploited to improve outcomes, the response to RT revealed in this analysis is novel, with clear opposing signal signatures identified between survival groups with differential alterations in RT-specific pathways including UV response UP and DN unfolded protein response (UPR) (**Supplemental Figure 2**). Previous research has shown that radiosensitization may be mediated by reduced expression of proteins critical to radioresponse (59), however considering the large number of signals present in Hallmark UV response_DN (144 genes) and UV response _UP (158 genes) and the dynamic nature of the protein signal, conclusions here may be difficult to attribute to any one or even a small set of proteins. UV response_UP was elevated in the intermediate group, and UV response_DN and UPR were decreased in the lowest survival group. This could be consistent with tumor heterogeneity and signaling ambivalence and may be connected to stem cell burden in the group of patients with the lowest survival. The UPR response is defined by a complex balance between prosurvival and prodeath proteins and has been reported as engaged in glioma as a means of migration and proliferation in response to a stressed state that may involve hypoxia due to extensive cell proliferation (60). There is growing evidence that RT-induced K-RAS/ERK signaling activation elevates CD44 expression through downregulation of miR-202 and miR-185 expression, promoting SRC activation to drive cancer stemness and EMT as a pivotal mechanism to mesenchymal transition in GBM cells (61). Given this understanding of molecular mechanisms underlying the therapeutic response, the decrease in UPR and increase in KRAS signaling DN, captured in the poor survival group could be attributed to insensitivity to RT or chemotherapy-induced stress and/or a decrease in prodeath related proteins that may mediate resistance to therapy which could be expoited with adjuvant targetted therapy. In summary clinical survival groups have unique proteomic serum signatures that uncovered a delicate balance between resistance, stem cell sustainment, apoptosis and the leverage of immunometabolic pathways which could be targeted therapeutically irrespective of oncogenic mutation status.

### Protein analysis

Nine proteins were significantly differentially expressed between groups and associated with survival, 1 of which was also statistically significant on Kaplan Meier analysis (CEACAM16). CEACAM 16, while novel in its association with GBM, is a member of the carcinoembryonic antigen family with several carcinoembryonic antigen-associated cell adhesion molecules having been associated with tumor infiltration, migration and invasion as well as mediators of immune function and cell adhesion (62). Recent evidence also suggests that CEACAMs are implicated in modulating dependent adhesion between glioblastoma initiating cells and surrounding cells via signaling through STAT3 (63) and are known for the association with hallmarks of cancer (involving stemness, surfaceome, ECMreceptor interaction, focal adhesion, platelet activation, the PI3KAkt pathway and invasion) all supported by evidence to have an association with prognosis in glioma (64–68) and in our serum proteome alteration analysis they connected to survival. The top altered protein in the lowest survival group was MGMT possibly in keeping with the known prognostic effect MGMT methylation status in GBM (42). While the alteration in these and other proteins is of significant interest, given their association with known hallmarks of cancer pathways, care should be taken in interpreting these results for directionality since gene signatures do not always correlate with proteomic expression and further analysis is needed to fully characterise the connection between MGMT promoter methylation status and MGMT protein expression in this cohort.

### Study limitations

The limitations of the study include the small sample size and retrospective nature of the study. 36% of the cohort received VPA concurrently on trial and the proteome response in this subcohort is potentially distinct which is the subject of future directions and separate analysis. MGMT methylation status was unknown in 37% of the cohort, and IDH mutation status was unavailable for all patients. The patients were diagnosed between 2005 and 2013 before measurement of IDH mutation status became part of the standard of care. In addition given that the tissue samples from these patients date to anywhere from 18 to 10 years ago performing this measurement is likely to be unreliable. We also note that the changes in protein expression, while statistically significant, are relatively small in magnitude. One uncertain aspect of the study is the extent to which the measured proteins come from cancer cells destroyed by the treatment vs. from surviving active cancer cells. Proteins from killed cells might not have much predictive value. This study provides a significant advance in this area, but more work needs to be done to validate and extend these results.

## Conclusion

We have demonstrated that the serum proteome exhibits biologically congruent alteration following standard of care management in GBM in a manner that connects to clinical survival and response to chemotherapy and radiation therapy. This is the first study examining the serum proteome pre and post treatment in GBM. The data presented here suggest that it may be possible to monitor GBM patient progression and response to treatment *via* the serum proteome since clinical survival groups follow different serum proteome alteration templates and this could facilitate improved individualized care. It is anticipated that both vaidation and superior clinical-proteomic classification is forthcoming as clinical and proteomic data acquisition and analysis advance in neuro-oncology.While individual protein signals may be insufficiently robust to classify outcomes at this time, promising signals are emerging and since clinical survival groups are associated with the proteome *via* known hallmark pathways in cancer, this could be exploited to advance new therapies. Future efforts are directed at defining the proteomic signal along specific clinical data streams, to hone in on potentially significant protein signals that are measurable and provide potentially druggable targets to advance outcomes.

## Data availability statement

The datasets presented in this study can be found as **Supplementary Material**.

## Ethics statement

The studies involving human participants were reviewed and approved by NCI NIH IRB. The patients/participants provided their written informed consent to participate in this study.

## Author contributions

AK: Conceptualization, Investigation, Data Curation, Supervision, Writing Original Draft Preparation, Visualization, Review and Editing, Project administration, Funding Acquisition;

MS: Data Curation, Methodology, Software, Investigation, Writing Original Draft Preparation, Visualization, Review and Editing; TN: Data Curation, Methodology, Software, Investigation, Writing Original Draft Preparation, Visualization, Review and Editing; QC: Methodology, Software, Investigation, Review and Editing; CY: Methodology, Software, Investigation, Review and Editing; YH: Methodology, Software, Investigation, Review and Editing; WJ: Data Curation, Software, Review and Editing; ET: Data Curation, Review and Editing; TZ: Data Curation, Review and Editing; MS: Data Curation, Review and Editing; MM: Data Curation, Review and Editing; US: Data Curation, Review and Editing; DM: Investigation, Supervision, Review and Editing, Project administration, Funding Acquisition; KC: Conceptualization, Investigation, Supervision, Review and Editing, Project administration, Funding Acquisition; All authors have read and agreed to the published version of the manuscript.

## Supplementary material

Supplemental file paired t-test against the MSigDB Hallmark, Canonical Pathways, GO Biological Process gene sets.

Supplemental file The blue module (637 proteins) and its Enrichr results for different databases.
