# Corrigendum: Glioblastoma survival is associated with distinct proteomic alteration signatures post chemoirradiation in a large-scale proteomic panel

**DOI:** 10.3389/fonc.2024.1348105

**Published:** 2024-03-13

**Authors:** Andra Valentina Krauze, Michael Sierk, Trinh Nguyen, Qingrong Chen, Chunhua Yan, Ying Hu, William Jiang, Erdal Tasci, Theresa Cooley Zgela, Mary Sproull, Megan Mackey, Uma Shankavaram, Daoud Meerzaman, Kevin Camphausen

**Affiliations:** ^1^Radiation Oncology Branch, Center for Cancer Research, National Cancer Institute, NIH, Bethesda, MD, United States; ^2^Computational Genomics and Bioinformatics Branch, Center for Biomedical Informatics and Information Technology, National Cancer Institute, NIH, Rockville, MD, United States

**Keywords:** glioma, radiation, proteomic, genomic, classification

In the published article

42. Hegi, M.E., et al., MGMT gene silencing and benefit from temozolomide in glioblastoma. *N Engl J Med*, 2005. 352(10): p. 997-1003

62. Beauchemin, N. and A. Arabzadeh, Carcinoembryonic antigen-related cell adhesion molecules (CEACAMs) in cancer progression and metastasis. *Cancer Metastasis Rev,* 2013. 32(3-4): p. 643-71.

63. Kaneko, S., et al., Ceacam1L Modulates STAT3 Signaling to Control the Proliferation of Glioblastoma-Initiating Cells. *Cancer Res,* 2015. 75(19): p. 4224-34.

were not cited in the article. The citations have now been inserted in **References**, *as reference numbers 42, 62, 63* and should read:

“Nine proteins were significantly differentially expressed between groups and associated with survival, 1 of which was also statistically significant on Kaplan Meier analysis (CEACAM16). CEACAM 16, while novel in its association with GBM, is a member of the carcinoembryonic antigen family with several carcinoembryonic antigen-associated cell adhesion molecules having been associated with tumor infiltration, migration and invasion as well as mediators of immune function and cell adhesion (62). Recent evidence also suggests that CEACAMs are implicated in modulating dependent adhesion between glioblastoma initiating cells and surrounding cells via signaling through STAT3 (63). The top altered protein in the lowest survival group was MGMT possibly in keeping with the known prognostic effect MGMT methylation status in GBM (42).”

The authors apologize for this error and state that this does not change the scientific conclusions of the article in any way. The original article has been updated.

In the published article, there was an error in **Figures 2**, **4**, **5**, **6**, **7**, **8** and **Table 3** as published. Some of the samples were incorrectly labelled and this affected 7 out of 82 patients in the study requiring the proteomic components of the analysis to be repeated. The corrected **Figures 2**, **4**, **5**, **6**, **7**, **8** and **Table 3** and their corrected captions below each figure appear below.

**Figure 2:**
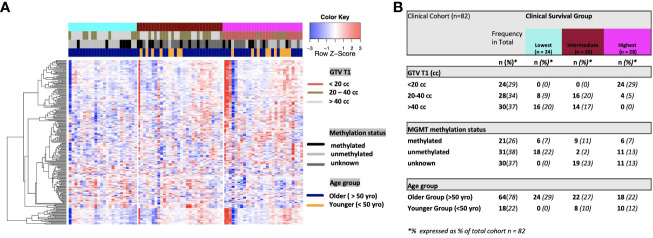
**(A)** Heat map of clinical clustering and proteomic signal following overall survival analysis based on GTV T1, MGMT methylation status, and age group with Cox proportional hazard p-values < 0.05. The dendrogram represents 221 significantly expressed proteins pre- vs. post-treatment. Unknown methylation status is represented by the color light grey. **(B)** Frequencies of clinical factors in the whole cohort and clinical survival group (GTV T1, MGMT methylation status, and age group) broken down by survival group n (expressed as % of total cohort n=82).

**Figure 4:**
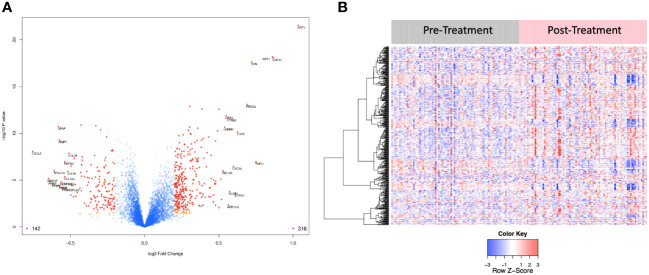
**(A)** Volcano plot of differentially expressed proteins pre vs. post treatment. Red dots represent the significant proteins that passed cut-off (|Log2FC| > 0.2 & FDR < 0.05) (458 proteins). Orange dots represent the proteins with |Log2FC| > 0.2 but not FDR < 0.05. Blue dots represent proteins that are not significant (i.e., did not pass either threshold). 316 proteins were up regulated, and 142 proteins were downregulated. The top 15 proteins that decreased in value (left aspect of plot) and those that increased in value (right aspect of plot) based on |FC| were labeled with the identified proteins’ names. **(B)** Heat map representation of the expression levels of selected, differential expressed proteins (N=458) pre vs. post treatment.

**Figure 5:**
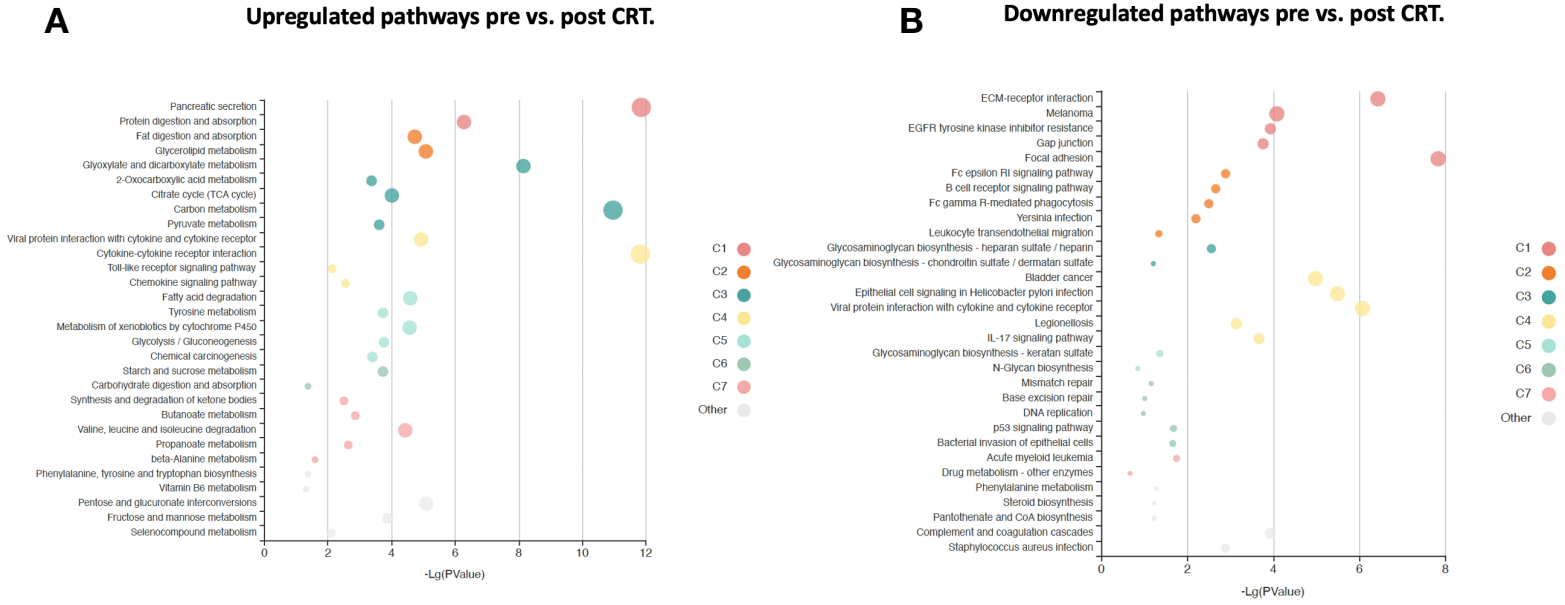
KOBAS bubble plot of KEGG pathways enriched in the set of significantly differentially expressed proteins between pre-and post-treatment based on a paired t-test. **(A)** Upregulated genes (FDR < 0.05). **(B)** Downregulated genes (FDR < 0.05). The bubble size reflects the KOBAS hypergeometric test p-value broken into the following ranges (smallest to largest): [0.05,1], [0.01,0.05), [0.001,0.01), [0.0001,0.001), [1e-10,0.0001), [0,1e-10). Colors reflect clusters of related pathways. Clusters of pathways are determined by creating edges if the Jaccard Index between pathways is larger than 0.35 and then clustering using the Infomap algorithm.

**Figure 6:**
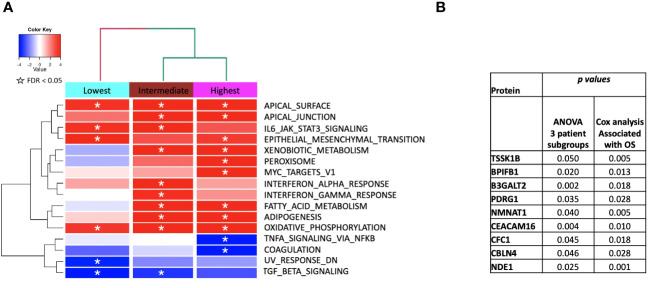
**(A)** ssGSEA2.0 associations with Hallmark pathways for proteins differentially expressed within patient subgroups. Paired t-tests were run within each of the patient subgroups from Figure 1. These results were fed into ssGSEA2.0 and significant pathways (FDR < 0.05) in at least one of the three subgroups were selected. The heatmap shows the GSEA normalized enrichment score. **(B)** The 9 proteins statistically significant across the three subgroups (p value (ANOVA) < 0.05) and associated with OS in Cox analysis (p < 0.05). Kaplan Meier analysis for statistically significant proteins is shown inSupplemental Figure 1.

**Figure 7:**
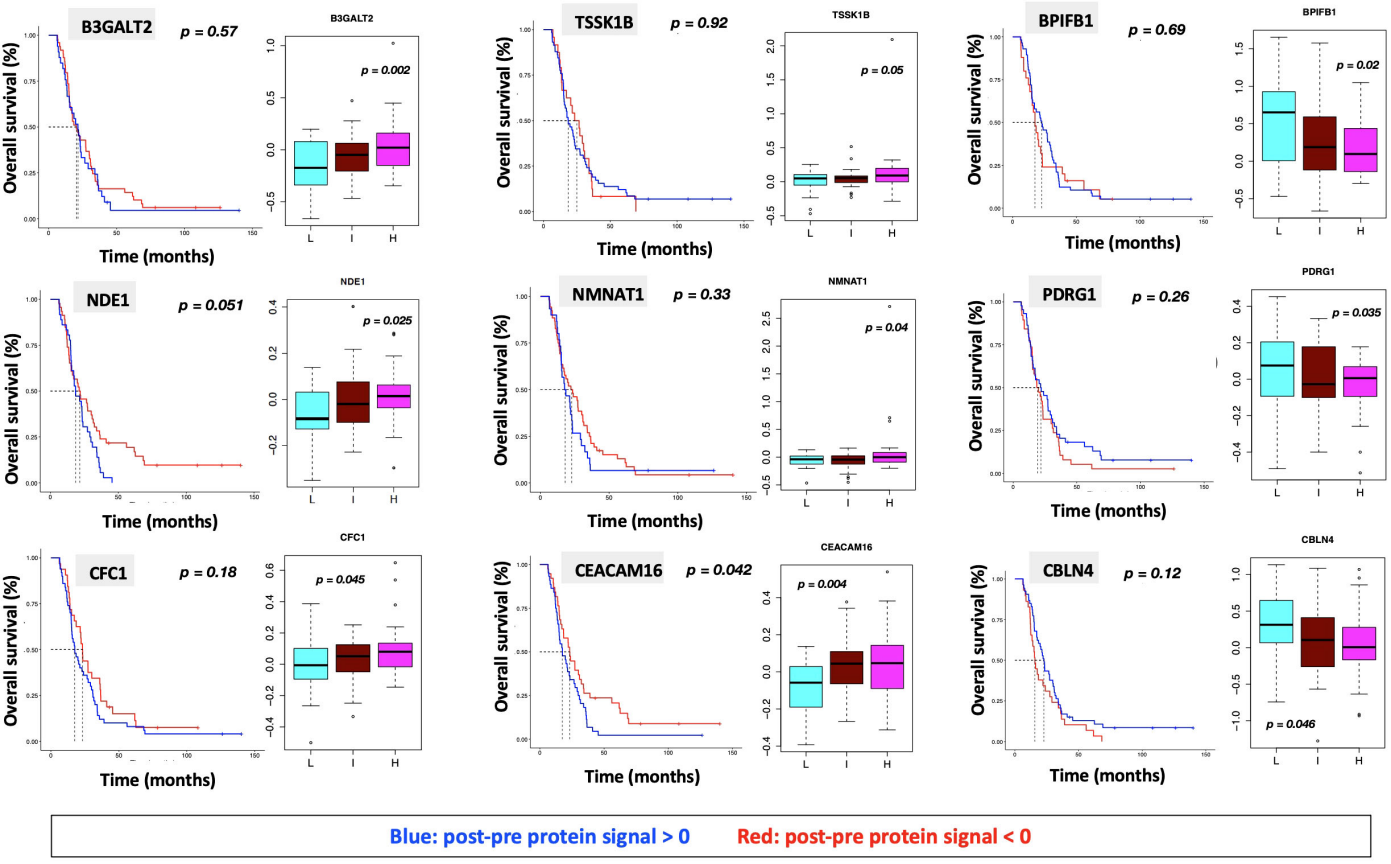
Overall survival analysis for 9 proteins statistically significant across the three subgroups (p value (ANOVA) < 0.05) and associated with OS in Cox analysis (p < 0.05) with associated protein signal box plots.

**Figure 8:**
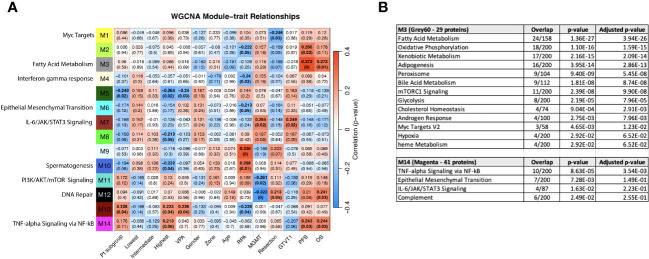
**(A)** WGCNA Protein module-clinical trait relationships heatmap for post-pre data with clinical features. The WGCNA protein modules are labeled with the MSigDB Hallmark pathways with the lowest adjusted p-value according to Enrichr. The M3 (Grey60) and M14 (Magenta) modules significantly correlate to overall survival and progression free survival. M7 (Brown) significantly correlated with MGMT methylation status and GTVT1. The top numbers indicate correlation coefficients, numbers in brackets indicate p-values. Bold indicate cells with p ≤ 0.05. **(B)** GO Biological Processes that are associated with the M3 and M14 modules according to Enrichr. MGMT methylation status (1= methylated, 2 = unmethylated).

**Table 3:** The top ten proteins based on p-value identified in a paired t-test comparing pre- and post-CRT expression for each patient subgroup.

Lowest Survival Subgroup (n=24)			Intermediate Survival Subgroup (n=30)			Highest Survival Subgroup (n=28)		
Symbol	p-value	Log2FC	Symbol	p-value	Log2FC	Symbol	p-value	Log2FC
MGMT	7.29E-06	-0.627	*SFN*	2.74E-11	0.808	**KRT5**	9.83E-10	1.128
**KRT5**	1.04E-05	0.986	**KRT5**	8.01E-10	0.993	**KRT1**	4.70E-08	0.996
GDF15	3.03E-05	0.638	**KRT1**	1.80E-09	0.827	*SFN*	2.40E-07	0.775
EDA2R	3.34E-05	0.176	**GDF15**	4.59E-09	0.980	**GDF15**	3.26E-07	0.944
GFAP	3.56E-05	-0.681	FAS	7.64E-09	0.339	**SERPIND1**	2.73E-06	-0.264
**KRT1**	1.14E-04	0.749	CPB1	3.14E-08	0.781	**ACP7**	4.27E-06	-0.213
ILR1	1.21E-04	0.375	*PRSS1*	3.53E-08	0.516	**HMX2**	4.94E-06	-0.085
TAGLN	1.60E-04	0.318	CTRB2	8.42E-08	0.689	**GUCA1B**	5.45E-06	-0.171
*PRSS1*	1.93E-04	0.302	CBR3	9.27E-08	0.313	**PRSS27**	6.00E-06	-0.264
PRSS2	3.02E-04	0.518	BOC	1.23E-07	0.587	**RAB26**	6.19E-06	-0.171

Gene symbols appearing in all three subgroups are shown in bold, those appearing in two subgroups are shown in italics. Genes symbols whose expression dropped post treatment are underlined. FC, fold change.

The authors apologize for this error and state that this does not change the scientific conclusions of the article in any way. The original article has been updated.

In the published article, there was an error in Supplementary Figures 1, 2, Table 1 and Supplementary Files 1 and 2. Some of the samples were incorrectly labelled and this affected 7 out of 82 patients in the study requiring the proteomic components of the analysis to be repeated. The correct material statement appears below.

The authors apologize for this error and state that this does not change the scientific conclusions of the article in any way. The original article has been updated.

In the published article, there was an error. Some of the samples were incorrectly labelled and this affected 7 out of 82 patients in the study requiring the proteomic components of the analysis to be repeated.

A correction has been made to **Abstract,** *Subsection: Results*. This sentence previously stated:

“ 3 clinical clusters of patients with differential survival were identified. 389 significantly DEPs pre- vs. post-treatment, 284 upregulated, 105 downregulated emerged including several pathways relevant to cancer metabolism and progression. The worst survival group (median OS 13.2 months) was associated with DEPs affiliated with proliferative pathways and distinct oppositional response (including RT) as compared to better-performing groups (intermediate, median OS 22.4 months; highest, median OS 28.7 months). Opposite signaling patterns across multiple analyses in several pathways (notably fatty acid metabolism, NOTCH, TNFα via NF-κB, Myc target V1 signaling, UV response, unfolded protein response, peroxisome, and interferon response) were distinct between clinical survival groups and supported by WGCNA. 23 proteins were statistically significant for OS with 5 (NETO2, CST7, SEMA6D, CBLN4, NPS) supported by KM.”

The corrected sentence appears below:

“3 clinical clusters of patients with differential survival were identified. 458 significantly DEPs pre- vs. post-treatment, 316 upregulated, 142 downregulated emerged including several pathways relevant to cancer metabolism and progression. The worst survival group (median OS 13.2 months) was associated with DEPs affiliated with proliferative pathways and distinct oppositional response (including RT) as compared to better-performing groups (intermediate, median OS 22.4 months; highest, median OS 28.7 months). Opposite signaling patterns across multiple analyses in several pathways (notably fatty acid metabolism, TNFα via NF-κB, Myc target V1 signaling, UV response, unfolded protein response, peroxisome, and interferon response) were distinct between clinical survival groups and supported by WGCNA. 9 proteins were statistically significant for OS with 1 (CEACAM16) supported by KM.”

A correction has been made to **Materials and Methods**, [*Subsection: Survival associated protein signal analysis***]**. This sentence previously stated:

“Univariate Cox modeling using the post-pre log2-transformed RFU values was performed to identify proteins associated with OS. ANOVA was performed to identify differentially expressed proteins (DEPs) between the three clinical subgroups with 23 significant proteins (p < 0.05) in both tests (2 of which are part of a complex). KM analysis on these 23 proteins was performed using the survminer package by separating patients into two groups with log2FC greater than or less than zero.”

The corrected sentence appears below:

“Univariate Cox modeling using the post-pre log2-transformed RFU values was performed to identify proteins associated with OS. ANOVA was performed to identify differentially expressed proteins (DEPs) between the three clinical subgroups with 9 significant proteins (p < 0.05) in both tests. KM analysis on these 9 proteins was performed using the survminer package by separating patients into two groups with log2FC greater than or less than zero.”

A correction has been made to **Results**, [*Subsection: Differential protein expression in serum following CRT reveals pathways relevant to cancer both up and downregulated***]**. This sentence previously stated:

“The log2-fold change values ranged from 1.02 to -0.83, with 1847 proteins having an FDR less than 0.05 with 389 proteins having an |Log2FC| >= 0.2 (284 upregulated, 105 downregulated, **Figure 4**). The significantly up- and downregulated genes were entered into the KOBAS server, which performs gene set enrichment analysis using the hypergeometric test and generates plots (27). **Figure 5** shows that pathways relevant to cancer, such as the Ras, MAP kinase, NOTCH, and Hippo signaling pathways and metabolic pathways, are upregulated post CRT according to KOBAS. Various immune-related pathways are downregulated. The colors in **Figure 5** are clusters of pathways with overlapping genes. The fact that multiple related dark red pathways are up or downregulated together suggests that the differential expression measures a meaningful biological signal and is not artifactual.”

The corrected sentence appears below:

“The log2-fold change values ranged from 1.04 to -0.75, with 2298 proteins having an FDR less than 0.05 with 458 proteins having an |Log2FC| >= 0.2 (316 upregulated, 142 downregulated, **Figure 4**). The significantly up- and downregulated genes were entered into the KOBAS server, which performs gene set enrichment analysis using the hypergeometric test and generates plots (27). **Figure 5** shows that pathways relevant to cancer, such as the Ras, MAP kinase and NOTCH signaling pathways and various metabolic pathways, are upregulated post CRT according to KOBAS. Overall various metabolic pathways are upregulated, and immune-related pathways are downregulated. The colors in **Figure 5** are clusters of pathways with overlapping genes. The fact that multiple related pathways are up or downregulated together suggests that the differential expression measures a meaningful biological signal and is not artifactual.”

A correction has been made to **Results**, [*Subsection: GBM survival groups are associated with differential signaling pathways and serum protein expression***]**. This sentence previously stated:

“**Figure 6** shows the identified MSigDB Hallmark pathways with an FDR < 0.05 (**Figure 6A**), including KRAS signaling, Notch signaling, heme metabolism, angiogenesis, unfolded protein response (UPR), UV response up/down, Interferon alpha and gamma response, glycolysis, peroxisome, Myc targets V1 and TNFα signaling via NF-κB.”

The corrected sentence appears below:

“**Figure 6** shows the identified MSigDB Hallmark pathways with an FDR < 0.05 (**Figure 6A**), including Apical Junction, IL-6/JAK/STAT3 Signaling, Epithelial Mesenchymal Transition, Xenobiotic Metabolism, Fatty acid metabolism, TNF-alpha Signaling via NF-kB, Interferon alpha and gamma response, UV response down.”

A correction has been made to **Results**, [*Subsection: GBM survival groups are associated with differential signaling pathways and serum protein expression***]**. This sentence previously stated:

“The lowest survival group is significantly associated with increased signaling via Notch, KRAS, and heme metabolism and a decrease in signaling pathways related to angiogenesis, UPR, and UV response down (**Figure 6A**).”

The corrected sentence appears below:

“The lowest survival group lacks associations with metabolic pathways that distinguish intermediate and higher survival groups (**Figure 6A**).”

A correction has been made to **Results**, [*Subsection: GBM survival groups are associated with differential signaling pathways and serum protein expression***]**. This sentence previously stated:

“Similar associations with Notch signaling and pathways in cancer (lowest subgroup) and apoptosis (intermediate subgroup) and peroxisome (highest subgroup) were noted when examining enriched KEGG pathways Supplemental Table 1).”

The corrected sentence appears below:

“Similar associations with pathways in cancer were noted when examining enriched KEGG pathways (Supplemental Table 1).”

A correction has been made to **Results**, [*Subsection: GBM survival groups are associated with differential signaling pathways and serum protein expression***]**. This sentence previously stated:

“We identified 23 proteins (two of which are part of a complex) that were statistically significant across the three subgroups via ANOVA (p-value < 0.05) and also associated with OS in a univariate Cox model (p < 0.05) (**Figure 6B**). Five of these were also statistically significant upon Kaplan-Meier analysis (NPS, NETO2, SEMA6D, CBLN4, CST7) (**Figure 6B**). These five proteins all have plausible connections to relevant pathways; however, box plots of their expression demonstrate significant overlap in expression between patient subgroups, indicating they are unlikely to be effective as prognostic indicators. (**Figure 7**). Paired t-tests within the clinical subgroups (top 10 proteins emerging show in **Table 3**) reveal distinct proteins between the survival groups with respect to fold change and p values with proteins presetn in all groups (KRT5, KRT1, SFN, GDF15) and others particularly significant in others (CBR3, BOC, FAS and the cancer cell metabolism genes PNLIPRP1 and CPB1 in lowest survival group). Overall most of the proteins are elevated post treatment as compared to prior to treatment and while p values are statistically significant, FDRs are only significant for the top 8 and top 1 proteins in subgroups 2 and 3 (**Table 3**).”

The corrected sentence appears below:

“We identified 9 proteins that were statistically significant across the three subgroups via ANOVA (p-value < 0.05) and also associated with OS in a univariate Cox model (p < 0.05) (**Figure 6B**). One was also statistically significant upon Kaplan-Meier analysis (CEACAM16). These nine proteins all have plausible connections to relevant pathways; however, box plots of their expression demonstrate the significant overlap between patient subgroups, indicating they are unlikely to be effective as prognostic indicators. (**Figure 7**). Paired t-tests within the clinical subgroups (top 10 proteins emerging shown in **Table 3**) reveal distinct proteins between the survival groups with respect to fold change and p values with proteins present in all groups (KRT5, KRT1, GDF15) and others, particularly significant in others (MGMT in lowest survival group). Overall, most of the proteins are elevated post treatment compared to prior to treatment, and while p values are statistically significant, FDRs are only significant for the top 2 proteins in the lowest survival subgroup while highly significant in the intermediate and high survival subgroups (**Table 3**).”

A correction has been made to **Results**, [*Subsection: Association of clinical characteristics and survival groups with differential protein expression in serum reveals module clinical trait relationships correlating with progression free and overall survival***]**. This sentence previously stated:

“The most significant associations with OS and PFS were with modules M5 (dark red) and M6 (dark turquoise) (**Figure 8A**). The GO biological processes that are associated with the M5 and M6 modules according to Enrichr are shown in **Figure 8B**. The dark red module (M5) has genes related to immune response, apoptosis, and Il-6/JAK/STAT signaling. The dark turquoise (M6) module contains genes associated with angiogenesis and cell proliferation via KRAS signaling down.”

The corrected sentence appears below:

“The most significant associations with OS and PFS were with modules M3 (Grey60) and M14 (Magenta) (**Figure 8A**). The GO biological processes associated with the M3 and M14 modules, are shown in **Figure 8B**. The grey60 (M3) and magenta (M14) modules contain genes associated with angiogenesis and cell proliferation via KRAS signaling down.”

A correction has been made to **Results**, [*Subsection: Association of clinical characteristics and survival groups with differential protein expression in serum reveals module clinical trait relationships correlating with progression free and overall survival***]**. This sentence previously stated:

“Consistent with **Figure 6A**, both “Pt subgroup” and “Lowest” are significantly correlated with module M11, which is enriched for the Hallmark fatty acid metabolism pathway.”

The corrected sentence appears below:

“Consistent with **Figure 6A**, both “Pt subgroup” and “Highest” are significantly correlated with modules M5 (Dark green) and M13 (Dark Red) while the “Highest” subgroup is associated with module M14 (Magenta) which is enriched for TNF-alpha Signaling via NF-kB, Epithelial Mesenchymal Transition, IL-6/JAK/STAT3 Signaling, Complement, Apical Junction, Xenobiotic Metabolism, Apoptosis, Unfolded Protein Response, KRAS Signaling Up and UV Response Up.”

A correction has been made to **Results**, [*Subsection: Association of clinical characteristics and survival groups with differential protein expression in serum reveals module clinical trait relationships correlating with progression free and overall survival***]**. This sentence previously stated:

“The highest survival subgroup has an inverse relationship with module M8 (tan), which is associated with TNFα signaling via NF-κB (again similar to **Figure 6A**), and a similar relationship is seen with VPA administration. The highest survival subgroup is enriched in the proportion of VPA treated patients (14/28) (50%) vs. subgroup 1 (5/24) (21%) and subgroup 2 (10/30) (33%). Modules 7, 9, and 10 are all associated with EMT, with oppositional trends between the lowest and highest survival groups. Module 9 (blue) is negatively associated with MGMT status (with a low correlation coefficient of -0.25). The blue module (637 proteins) and its Enrichr results for different databases are available as Supplemental Files.”

The corrected sentence appears below:

“The highest survival subgroup is enriched in the proportion of VPA treated patients (14/28) (50%) vs. subgroup 1 (5/24) (21%) and subgroup 2 (10/30) (33%). Module 11 (PI3K/AKT/mTOR signaling) and 12 (DNA repair) are negatively associated with MGMT status (with a low correlation coefficient of -0.26 and -0.32 respectively) and positively associated with M7 (Brown - which is associated with MGMT status and GTVT1), with a correlation coefficient of 0.25. The Grey60, Magenta and Brown modules and their Enrichr results are available as Supplemental File 2.”

A correction has been made to **Discussion,** [*Subsection: Pathway analysis***]**. This sentence previously stated:

“The group with the lowest survival distinguished by elevation in Notch signaling, KRAS signaling, and heme metabolism and a decrease in UPR, angiogenesis, and UV_response_DN in the Hallmark genesets, all similarly noted in KEGG and WGCNA, reveals a pathway signature consistent with poor prognosis in glioma via increased proliferation, hypoxia, enrichment in glioma stem cells, and radiation resistance via Notch signaling (47), IL-6_JAK_STAT3 (48), and UPR (49).”

The corrected sentence appears below:

“The group with the lowest survival, distinguished by UV_response_DN and a lack of association with metabolic pathways observed in the intermediate and higher survival groups in the Hallmark genesets, all similarly noted in KEGG and the WGCNA analysis, reveal a pathway signature consistent with poor prognosis in glioma via increased proliferation, hypoxia, enrichment in glioma stem cells, and radiation resistance via Notch signaling (47), IL-6_JAK_STAT3 (48, 49).”

A correction has been made to **Discussion,** [*Subsection: Protein analysis***]**. This sentence previously stated:

“Twenty-three proteins were significantly differentially expressed between groups and associated with survival, 5 of which were also statistically significant on Kaplan Meier analysis (NETO2, CST7, SEMA6D, CBLN4, NPS). These are all known for association with hallmarks of cancer (NETO2 - STAT/JAK/PI3K; CST7 - CST7 stemness; SEMA6D – surfaceome; CBLN4 - ECM-receptor interaction, focal adhesion, platelet activation, and the PI3K-Akt pathway; NPS - invasion) and some are supported by evidence to have an association with prognosis in glioma (62–66) and in our serum proteome alteration analysis they connected to survival. The top altered protein in the lowest survival group was CBR3 (Carbonyl reductase 3), a metabolically connected protein and downstream target of LGR5, a marker of poor prognosis in GBM required for survival of stem cell like cells (67). One of the top altered proteins in the highest survival group, CD163 has already been validated as a key immune marker in a recent proteogenomic analysis (4). While the alteration in these and other proteins is of interest, given their association with known hallmarks of cancer pathways, care should be taken in interpreting these results with respect to directionality since gene signatures do not always correlate with proteomic expression.”

The corrected sentence appears below:

“Nine proteins were significantly differentially expressed between groups and associated with survival, 1 of which was also statistically significant on Kaplan Meier analysis (CEACAM16). CEACAM 16, while novel in its association with GBM, is a member of the carcinoembryonic antigen family with several carcinoembryonic antigen-associated cell adhesion molecules having been associated with tumor infiltration, migration and invasion as well as mediators of immune function and cell adhesion (62). Recent evidence also suggests that CEACAMs are implicated in modulating dependent adhesion between glioblastoma initiating cells and surrounding cells via signaling through STAT3 (63) and are known for the association with hallmarks of cancer (involving stemness, surfaceome, ECM-receptor interaction, focal adhesion, platelet activation, the PI3K-Akt pathway and invasion) all supported by evidence to have an association with prognosis in glioma (64–68) and in our serum proteome alteration analysis they connected to survival. The top altered protein in the lowest survival group was MGMT possibly in keeping with the known prognostic effect MGMT methylation status in GBM (42). While the alteration in these and other proteins is of significant interest, given their association with known hallmarks of cancer pathways, care should be taken in interpreting these results for directionality since gene signatures do not always correlate with proteomic expression and further analysis is needed to fully characterise the connection between MGMT promoter methylation status and MGMT protein expression in this cohort.”

The authors apologize for this error and state that this does not change the scientific conclusions of the article in any way. The original article has been updated.

