# Supplementary figures and images for: Glioblastoma survival is associated with distinct proteomic alteration signatures post chemoirradiation in a large-scale proteomic panel
Source: Front Oncol. 2023 Aug 10;13:1127645. doi: 10.3389/fonc.2023.1127645 (PMC10448824; doi:10.3389/fonc.2023.1127645)

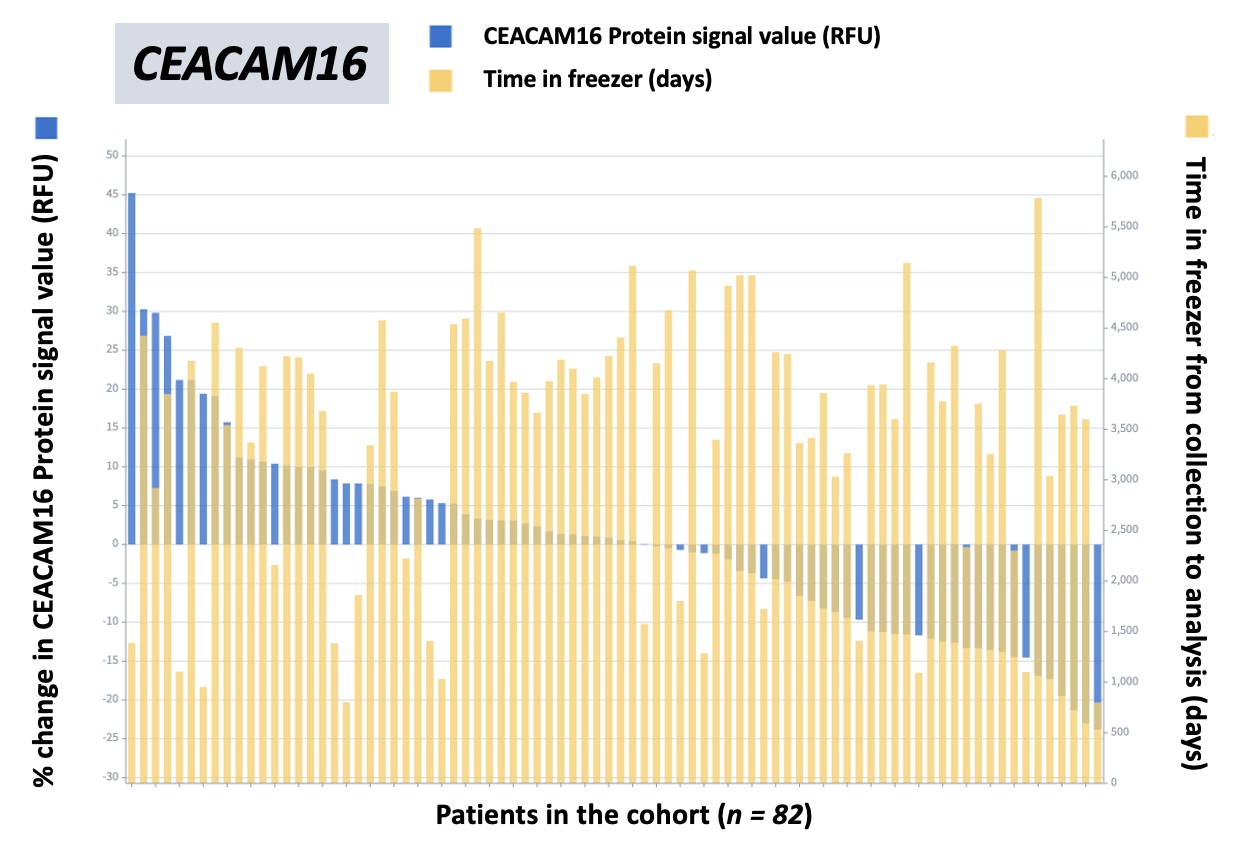

Supplement: Supplemental file — paired t-test against the MSigDB Hallmark, Canonical Pathways, GO Biological Process gene sets. [file Image_1.jpeg]

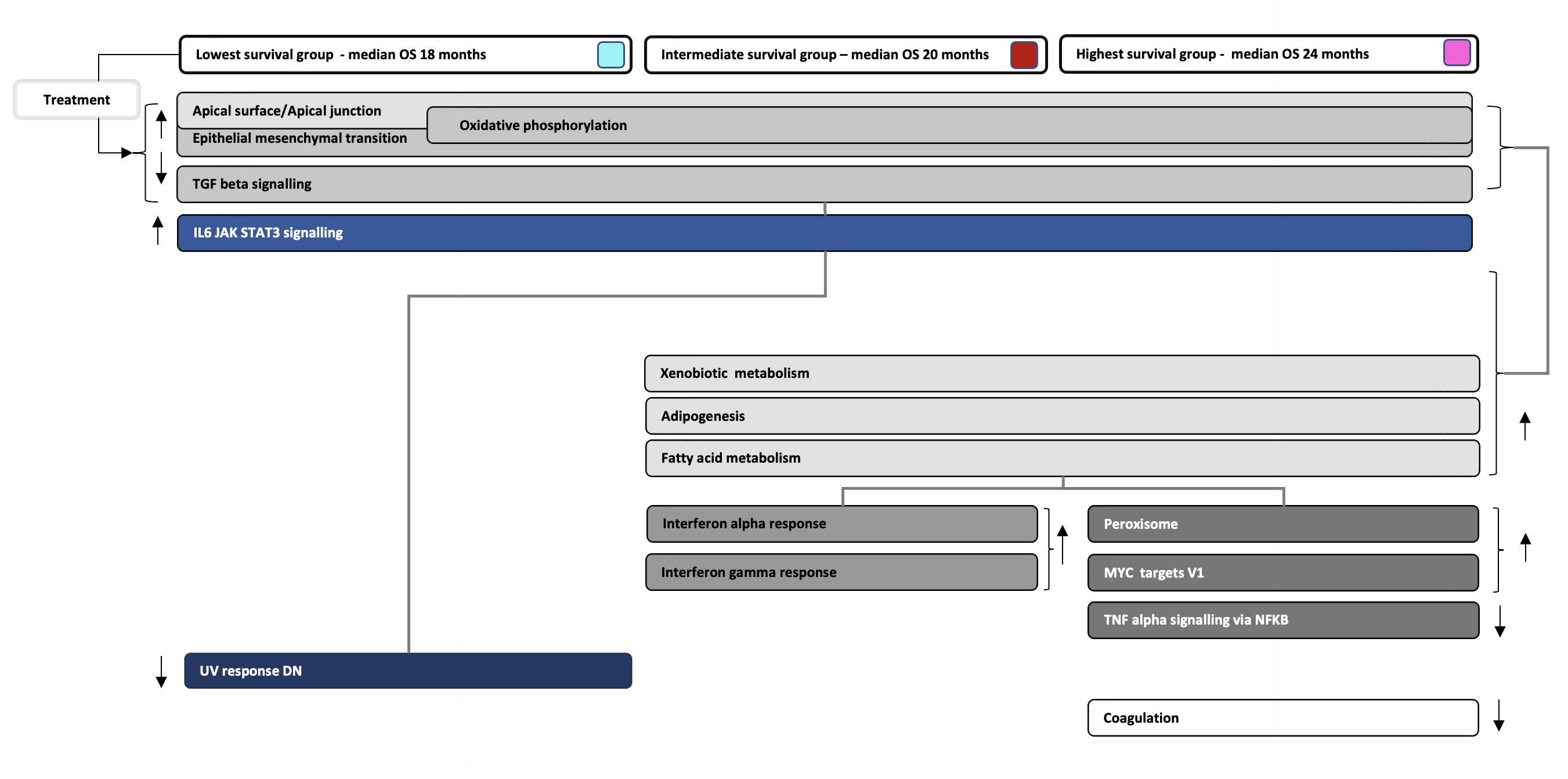

Supplement: Supplemental file — The blue module (637 proteins) and its Enrichr results for different databases. [file Image_2.jpeg]

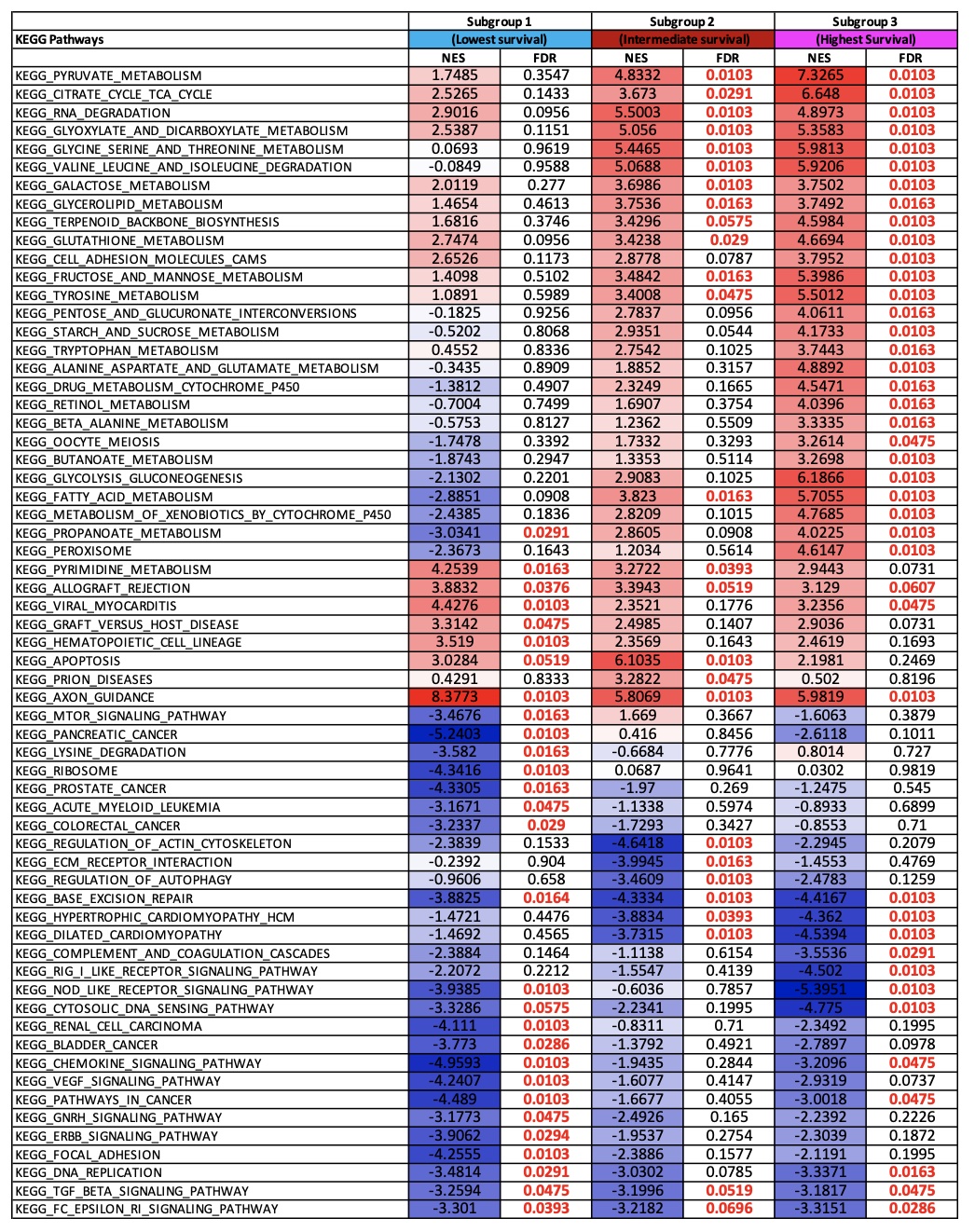

Supplement: Supplementary file 5 [file Image_3.jpeg]
